# Revision Arthroplasty for Bilateral McKee-Farrar Hip Prostheses 48 Years Following Implantation

**DOI:** 10.7759/cureus.44465

**Published:** 2023-08-31

**Authors:** Anirudh Sharma, Santosh Bindumadhavan, Sandeep Jiwanmall, Jayteja Killampalli, Vijay Killampalli

**Affiliations:** 1 Trauma and Orthopaedics, North West Anglia NHS Foundation Trust, Huntingdon, GBR; 2 Pathology, Sri Ramachandra Institute of Higher Education and Research, Chennai, IND

**Keywords:** metal-on-metal hip, arthroplasty, hip implants, revision hip arthroplasty, hip arthroplasty

## Abstract

The McKee-Farrar hip prosthesis gained popularity in the 1960s and was one of the first widely used prostheses to employ a metal-on-metal design. Eventually, it laid the framework for the development of second and third-generation hip replacement prostheses. In time, the McKee-Farrar prosthesis was found to have high rates of early aseptic loosening and fell out of favor, especially with the development of the Charnley low-friction metal-on-polyethylene design.

We present an interesting case of a patient who underwent bilateral total hip arthroplasty with a McKee-Farrar hip prostheses at the young age of 28 years, in view of secondary hip osteoarthritis resulting from ankylosing spondylitis. The implants lasted approximately 48 years after initial implantation in this case, which is an unusually long survival of this prosthesis. He presented to us at the age of 76 years with groin pain and difficulty to weight-bear, worse on the right side. Significant osteolysis around the acetabular component was noted, greater on the right side. Infection was excluded, and the patient underwent staged revision bilateral hip replacements one year apart. Extraction of the femoral components on both sides was done with the aid of extended trochanteric osteotomies. For both revisions, uncemented acetabular revision shells (TMARS, Zimmer Biomet, Warsaw, Indiana) were used for the acetabulum and long uncemented diaphyseal engaging interlocked stems (Arcos ILS, Zimmer Biomet) were used for the femoral side. No complications were encountered during the procedures. The patient made excellent progress following the procedures with immediate weight-bearing, as tolerated, and physiotherapy input. No subsequent postoperative complications occurred till the time of the patient's death five years later from unrelated medical causes.

It is rare to encounter and revise the McKee-Farrar prosthesis in modern orthopaedic practice. This, to the best of our knowledge, is the longest-described survival of this prosthesis in literature.

## Introduction

McKee and Watson-Farrar worked on the design of a hip prosthesis beginning in the 1950s and eventually developed the McKee-Farrar prosthesis, which after several variations, was eventually standardized by 1965 and widely used thereafter [1]. This design employed metal-on-metal articulation and both components were fixed with bone cement. The components were made of vitallium, a cobalt-chromium-molybdenum alloy. The choice of this alloy was based upon its inert nature in situ, alongside low friction and wear. The acetabular component was designed to be lipped and have studs on the fixation surface. The rationale of the studs was to increase the contact surface with cement and ensure a minimum cement depth. The femoral component was based on the then-popular Thompson prosthesis. It had a collared design and curved stem. The two components were manufactured in pairs to form a match with identical numbers on each [1].

With time, aseptic loosening was found to be the major cause of failure and revision of this prosthesis [2,3]. As the Charnley low-friction hip arthroplasty gained popularity by the mid-1970s, the use of the metal-on-metal McKee-Farrar prosthesis declined in favour of metal-on-polyethylene bearings.

We present a case of a patient with ankylosing spondylitis, who underwent bilateral total hip replacement with the said prosthesis in 1968, in whom these lasted 48 years. This is, to our knowledge, the longest surviving McKee-Farrar hip prosthesis described in the literature.

## Case presentation

This patient presented to our institution at the age of 76 years, having had primary bilateral total hip replacements 48 years prior, at a tertiary hospital in the United Kingdom. These were done as a result of secondary hip osteoarthritis developing due to ankylosing spondylitis. He had multiple other co-morbidities at presentation, which included a total colectomy with ileostomy performed in his teenage years for ulcerative colitis. He also had aortic regurgitation, hypothyroidism, obesity with a body mass index of 32, and was self-catheterising for benign prostate hyperplasia.

**Figure 1:**
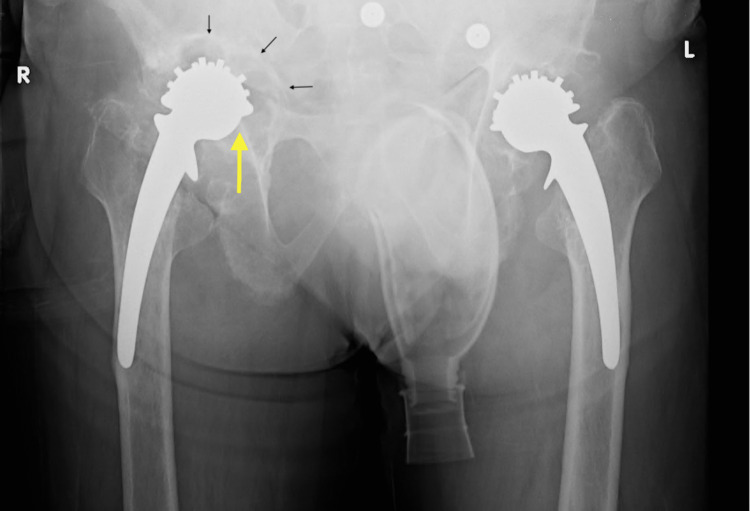
Radiographs on presentation showing retro-acetabular osteolysis (black arrows) with disruption of Kohler's line (yellow arrow) and ischial lysis on the right side with similar but less remarkable findings on the left

At presentation, he reported an insidious onset of pain in his hips for the last two years, the right side being much worse than the left. At the time, his mobility was limited to indoors, with a frame for support. X-rays of the pelvis with both hips (Figure 1) suggested significant retro-acetabular osteolysis in all three Gruen zones, greater on the right than the left. Proximal and medial migration of the cup was seen, with ischial lysis and disruption of Kohler’s line on the right side. Similar findings were present on the left side but with an intact Kohler’s line.

**Figure 2:**
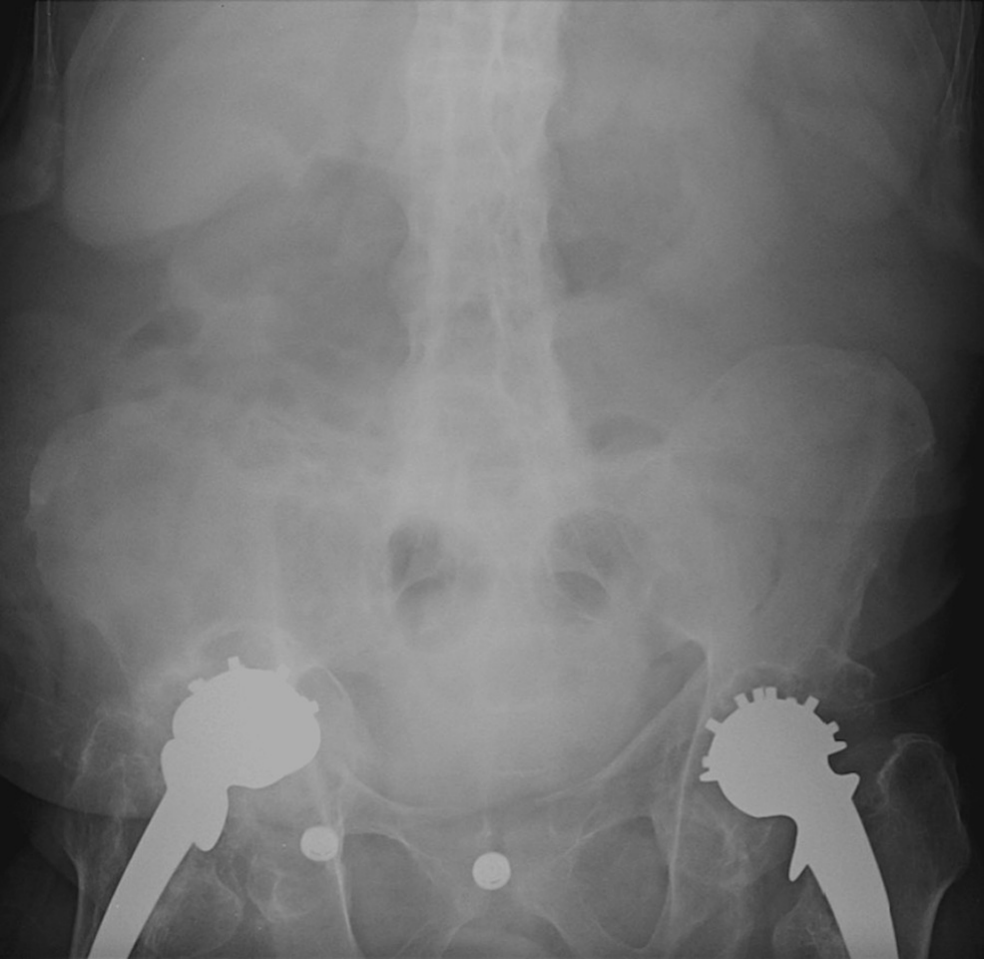
The patient had a completely ankylosed lumbar spine due to advanced ankylosing spondylitis

His lumbar spine was completely ankylosed from long-standing ankylosing spondylitis (Figure 2). He underwent further evaluation with a view to undertaking bilateral staged revision total hip replacements. Blood results revealed an erythrocyte sedimentation rate (ESR) of 18 mm/hr and a C-reactive protein (CRP) level of 14 mg/dL. Blood cobalt levels were found to be elevated at 133 mcg/L.

**Figure 3:**
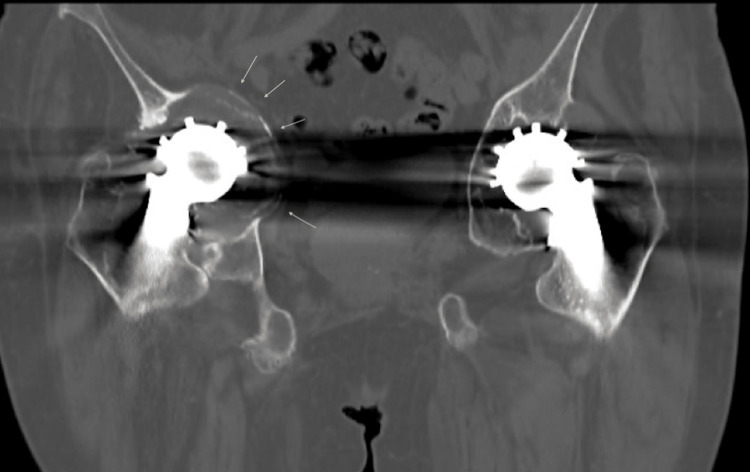
A coronal slice from the CT scan showing the extent of retro-acetabular osteolysis bilaterally, worse on the right side (see arrows)

A computed tomography (CT) scan confirmed the retro-acetabular osteolysis and the resulting protrusion worse on the right side (Figure 3). A bone scan was also performed, which did not suggest infection. A single-stage revision on both sides was planned and anaesthetic clearance was obtained.

**Figure 4:**
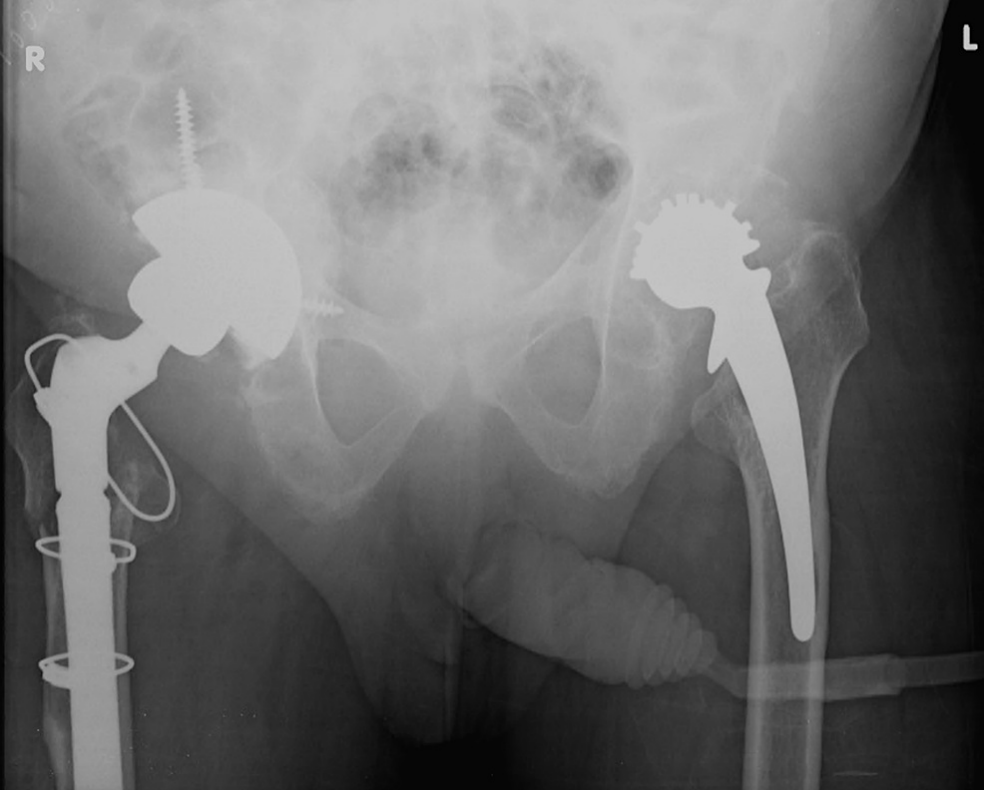
Postoperative pelvis X-rays after right revision total hip arthroplasty with an uncemented acetabular shell and a long uncemented diaphyseal engaging femoral stem

The right side was revised first, given the worse clinical and radiological symptoms on this side. A standard posterior approach was used for both sides. Intraoperatively, no signs of infection, metal staining of tissues, or pseudotumors were found. The abductor muscles were found atrophied. On the acetabular side, the defect was classified as Paprosky type 3b. Impaction bone grafting was used to address the defect, and reaming to 62 mm was performed. An uncemented trabecular metal revision acetabular shell (TMARS, Zimmer Biomet, Warsaw, Indiana) sized 66 was used with three screws for fixation. A TMARS revision liner for a 36 mm head was used. An extended trochanteric osteotomy (ETO) with a modified technique was used for femoral component extraction [4]. A long uncemented distally interlocking modular stem (Arcos ILS, Zimmer Biomet) was used, with cable grips to fix the ETO. Postoperative X-rays are shown in Figure 4.

**Figure 5:**
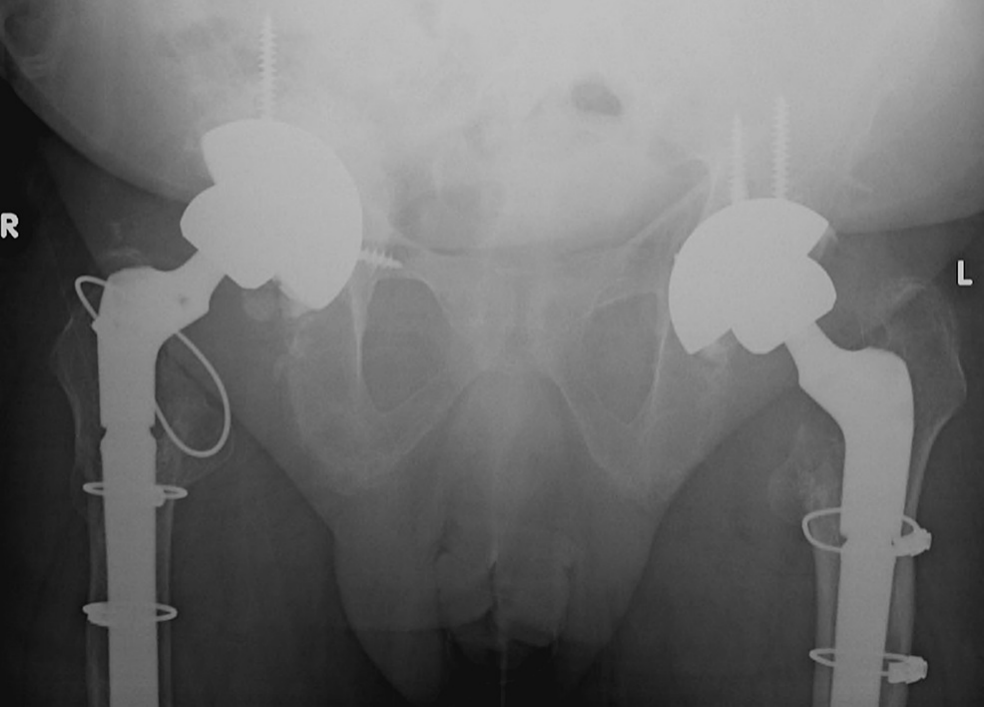
Final postoperative pelvis X-rays following left revision total hip arthroplasty, one year following revision on the right side

The patient had a slow but excellent recovery without any surgical complications and underwent left-sided revision one year later. On the left side, similar to the right, there was no evidence of metal staining or infection. The acetabulum was found to have poor support in the 11-3 o’clock position. A 60 mm uncemented trabecular metal shell (TMARS, Zimmer Biomet) was used and fixed with five screws. The femoral side was tackled in a similar manner to the right side. In neither revision were complications such as metal staining or pseudotumours encountered, despite a preoperative increase noted in blood cobalt levels. Final X-rays are shown in Figure 5. The patient made an excellent recovery and remained asymptomatic with his hips for the next five years. He achieved pain-free and stable hips bilaterally, although his mobility continued to be limited by his advanced ankylosing spondylitis and he continued to use a frame to mobilise. He died from medical causes unrelated to his surgeries five years later.

**Figure 6:**
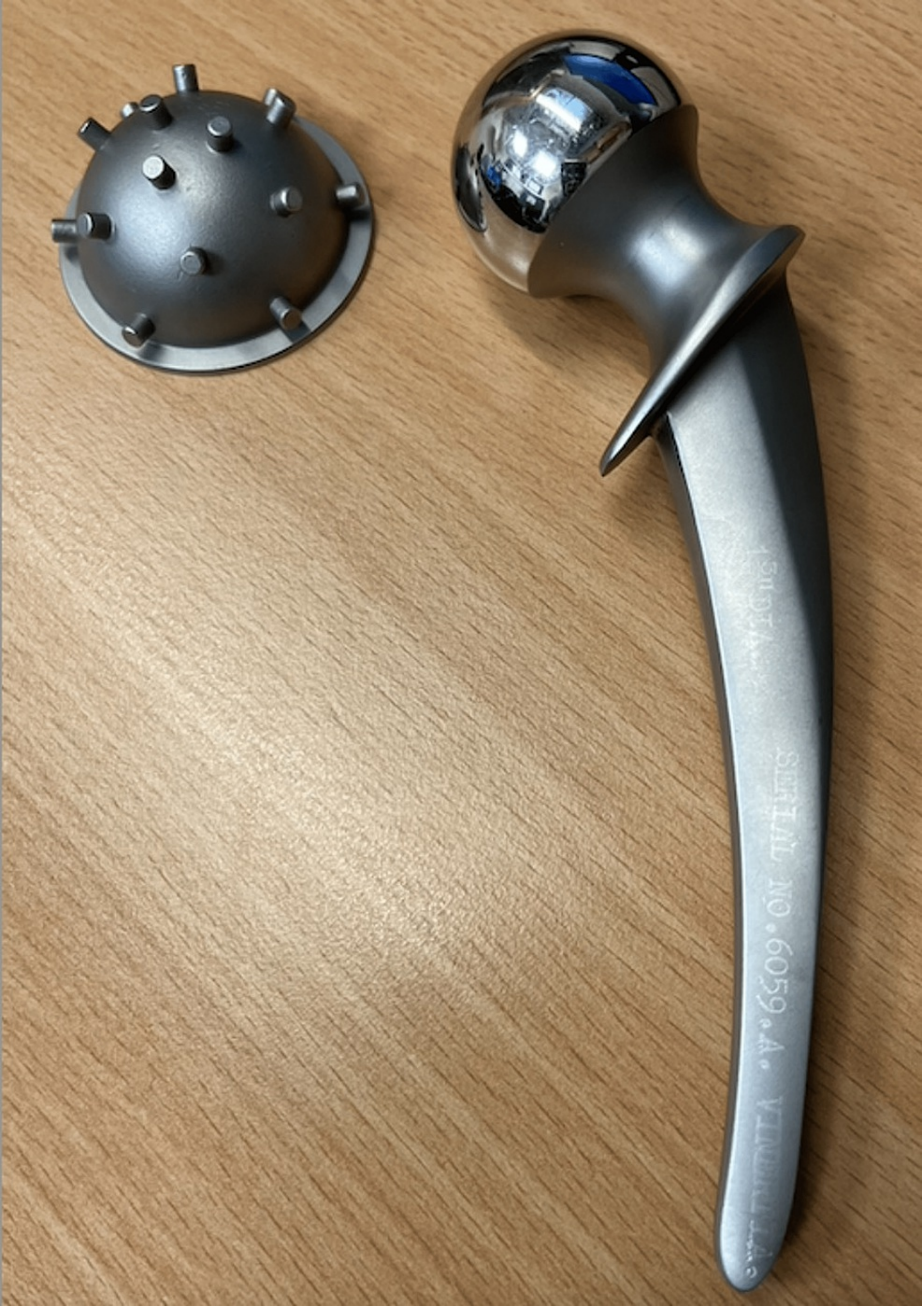
Extracted implant from the left side

An image of one of the extracted implants is shown in Figure 6.

## Discussion

To our knowledge following a literature search, this is the longest-known survival of the McKee-Farrar prosthesis. Long-term outcomes of this prosthesis have been reported in a number of studies [5-7]. In one of the largest studies looking at 808 McKee-Farrar hips implanted over an 8-year period between 1965 and 1973, survivorship at 20 years was found to be 27.5% [5]. Among causes of revision, 78% were as a result of aseptic loosening. Similarly, another prospective long-term study showed that the number of ‘good’ results after surgery fell drastically between the seventh and eighth postoperative year [6]. In another long-term study by Brown et al. [7], the survivorship of 123 prostheses at 20 years was found to be 84%, and at 28 years, it was 74%, which was significantly greater than that described in the August et al. study [5]. It has been shown that placement of the components to optimize hip biomechanics plays an important role in determining survival, which mainly includes medialisation of the hip centre and avoiding femoral stem varus [8]. Interestingly, the original technique described in the McKee-Farrar paper did not advocate for acetabular reaming, hence limiting the medialisation of the hip centre of rotation.

Clarke et al. in a case report described the clinico-radiological and histo-pathological findings of a McKee-Farrar prosthesis, which was functioning well at 30 years following implantation at the time of the patient’s death [9]. They found no cobalt-chromium debris and no metal debris in the tissues, concluding that the prosthesis functions well when tribological conditions are optimal. Szuszczewicz et al. describe a pattern of bilateral progressive pelvic osteolysis following bilateral implantation in their case report [10], which resembles the pattern of wear encountered in our case. In their report, one hip was revised after 13.5 years and was noted to have superomedial migration of the acetabular component with ischial osteolysis. In both the above reports, the authors performed a histo-pathological analysis and essentially found no convincing evidence of metal-wear debris as the cause of the osteolysis.

It is worth recognising that the failures of the McKee-Farrar prosthesis were largely due to shortcomings in design and fixation, rather than complications related to metal-on-metal articulation, which are known today [11]. The classically known complications of pseudotumor formation and metallosis were not well-described as complications with this prosthesis. While this first-generation prosthesis initiated an investigation into the issues of metal sensitivity as a cause for the loosening of these implants [12], it was later in the 1990s, when the phenomenon of ALVAL (aseptic lymphocytic vasculitis-associated lesions) was described [13].

While the pattern of failure seen in our case is similar to other reports with this implant [8,9], the low functional demand of our patient given his co-morbidities (ankylosing spondylitis, obesity etc.), is likely to have contributed to prolonged prosthesis survival. Although modern-day revision arthroplasty surgeons are unlikely to encounter this prosthesis, addressing the acetabular defects remains the main challenge when revising the McKee-Farrar prosthesis.

## Conclusions

The McKee-Farrar hip prosthesis was one of the first widely popularised hip implants that employed metal-on-metal articulation. Aseptic loosening with significant osteolysis in the acetabular region, rather than an adverse reaction to metal debris, was eventually found to be the most common cause of the failure of this implant. Our case report is unusual in that our patient had bilateral hip arthroplasty with this prosthesis, which lasted 48 years and is the longest-known survival of the McKee-Farrar hip prosthesis described in the literature. We also describe the challenges and techniques used to revise this prosthesis, which are centred around managing acetabular defects.
